# Dyspepsia among patients with chronic kidney disease: a cross sectional study

**DOI:** 10.1186/1755-7682-6-43

**Published:** 2013-10-20

**Authors:** Marcelo Rodrigues Bacci, Ethel Zimberg Chehter

**Affiliations:** 1 Department of General Practice, Faculdade de Medicina do ABC, Av. Principe de Gales n 821, Santo André CEP: 09060-650, Brazil; 2 Department of Gastroenterology, Faculdade de Medicina do ABC, Av. Principe de Gales n 821, Santo André CEP: 09060-650, Brazil; 3 Rua João Gross n 201 apto 81 bloco 1- Vila Gonçalves- São Bernardo do Campo, São Paulo CEP 09725-040, Brazil

**Keywords:** Dyspepsia, Functional dyspepsia, Gastroesophageal reflux disease, Chronic kidney disease, Hemodialysis

## Abstract

**Backgrounds:**

Dyspepsia is a condition that affects 25% of the U.S. population, and, when associated with pyrosis, its prevalence reaches 40%. Patients with chronic renal insufficiency not only present higher circulating levels of gastrin and gastric dysmotilty, but also make use of a great amount of drugs for the treatment of their comorbidities. This situation increases the chances of developing gastrointestinal symptoms. The aim of the current study is to evaluate the dyspeptic disease profile in patients undergoing hemodialysis, comparing them with chronic renal disease patients in conservative treatment and non-renal injury patients.

**Methods:**

This is a cross sectional study performed at the Gastroenterology department at Faculdade de Medicina do ABC in São Paulo, Brazil. Three groups were set aside according to the renal function levels calculated using the simplified MDRD formula. They answered three questionnaires that evaluated the presence of dyspepsia, functional dyspepsia and gastroesophageal reflux disease (GERD) associated with the performance of high digestive endoscopy.

**Results:**

A significant difference between the groups was observed concerning the renal function evaluated by the rates of creatinine clearance, creatinine and urea (p < 0.001). The rate of dyspepsia in the control group was higher than in patients with renal function alterations (p = 0.014). There was no difference between groups when it came to the presence of functional dyspepsia and GERD. However, there was a higher use of proton pump inhibitors in the hemodialysis group than in the other groups (p < 0.001).

**Conclusion:**

In the proposed model, there was no positive correlation between the worsening of the renal function and the presence of dyspepsia, functional dyspepsia and GERD. The longitudinal evaluation of hemodialysis patients is hampered by the high mortality rates in this group. There was a higher use of proton pump inhibitors, and it is believed that the dyspeptic symptoms are not acid-related.

## Backgrounds

Several definition proposals of dyspepsia have been made so far, and many of them converge to the difficulty to establish a differentiation between symptoms of gastric origin from the ones that are assumingly of the esophagus. Throughout time it has been stipulated that dyspepsia encompasses all the symptoms that are limited to the gastroduodenal region and not the esophagus [1].

In its last review on dyspepsia the American Gastroenterological Association (AGA) shows a prevalence rate of 25% for upper abdominal pain in the United States, and when associated with retroesternal burning this rate reaches nearly 40% [2]. In Brazil, there is a great variance among the studies and a precise percentage of the prevalence is uncertain.

According to the Montreal definition the gastroesophageal reflux disease (GERD) is a condition which develops when the reflux of the gastric contents causes troublesome symptoms and/or complications, like pyrosis and regurgitation, being associated or not with endoscopic alterations [3].

On the other hand, functional dyspepsia is defined as pain or discomfort in the upper abdomen with no structural or biochemical explanations for the symptoms, at least undetected by normal endoscopies, and symptoms that include pain, postprandial bloating, early satiety and an abdominal distention feeling over a period of 12 weeks for at least the past 6 months [4].

Like the dyspeptic syndrome, the chronic kidney disease (CKD) is highly prevalent in the world population. CKD is defined as the functional or structural alteration for a period of at least 3 months with health implications. CKD is classified according to its cause, glomerular filtration rate (GFR) levels and the albumin/creatinine ratio, being the two last ones prognostic indicators [5].

Patients with CKD, especially those who are on dyalisis, frequently complain of dyspeptic symptoms [6,7]. However, given the frequency of this complain, researchers have been working on models that establish a causal association with this symptomatology.

Patients with CKD present increased levels of gastrin, but this is not only due to the reduction in its clearance. There is a compensatory effect that occurs owing to the neutralization of the gastric juice as a consequence of the increase in ammonia levels [8].

The risk of digestive bleeding is also higher in such patients, especially in those with angiodysplasia lesions. Like the lesions mentioned, the association with hypertension, coronary insufficiency and congestive cardiac insufficiency also contribute to the bleeding [8].

The prevalence of dyspeptic symptoms in patients with renal diseases is extremely variable and its etiology is little defined because of the various risk factors for dyspepsia and GERD these patients have and undergo.

As a result of what was exposed, the aim of this study was not only to analyze the profile of dyspeptic symptoms in patients with CKD undergoing hemodialysis and in conservative treatment, but also to evaluate if a worse renal function constitutes a facilitating factor for the presence of functional dyspepsia, dyspepsia and gastroesophageal reflux.

## Method

### Study design

This is a cross-sectional study conducted between 2012 and 2013 in the Gastroenterology Department of the Faculdade de Medicina do ABC and in the Hemodyalisis center of Santo André. Patients were divided in three groups according to their glomerular filtration rates. Group 1 encompassed patients in hemodialysis; group 2 included those with CKD stages 1 to 4 according to the classification of CKD from the Kidney Disease Improving Global Outcomes; group 3 was formed by patients without kidney disease [5].

The study was submitted to and approved by the Research Ethics Committee of the Faculdade de Medicina do ABC under the number 067/2011. All subjects signed terms of informed consent before their participation.

### Patients

Eligible patients had to be between 18 and 70 years old and diagnosed with CKD stages 1 to 4 or in hemodialysis. Exclusion criteria included the presence of disease or clinical situation that could prevent patients from answering the questionnaires and patients who underwent renal transplant during the data collection phase. Patients with known gastric disease were not enrolled to answer the questionnaires in order to avoid selection byas.

Functional dyspepsia was defined as the absence of endoscopic alterations and the presence of symptoms established by the Rome III consensus, originated 6 months prior to the interview and present for at least 12 weeks [9].

According to the short form Leeds questionnaire, dyspepsia in its organic form was established as the presence of indigestion, pyrosis, nausea or regurgitation during the 2 months prior to the interview.

The evaluation for the presence of GERD was based on clinical parameters with the presence of pyrosis and regurgitation according to the Montréal definition, which was also adopted in this study. Endoscopic alterations were established as the presence of peptic ulcer (gastric or duodenal), erosive esophagitis and acute gastric mucosal injury. The other endoscopic findings were disregarded on account of the lack of causal link with dyspeptic disease.

CKD was defined as the presence of persistent alteration for at least 3 months with creatinine increase or textural alterations observed in imaging exams. The glomerular filtration rate determination was calculated by the application of the simplified MDRD formula, taking the values of serum creatinine during the interview into consideration [5,10].

Creatinine levels were obtained by the modified Jaffé reaction, and values of 1.3 mg/dl and 1.1 mg/dl were considered as altered for men and women respectively. Urea levels were determined by enzymatic colorimetric tests, and values above 50 mg/dl were considered altered. The evaluation of the presence of renal parenchyma alteration, whenever necessary, was performed by renal ultrasound.

The patients were submitted to an interview to answer the questionnaire that evaluated the functional dyspepsia according to the criteria adopted by the Rome Foundation (currently in its third review), which has been classifying gastrointestinal functional disorders since 1992. The Leeds questionnaire is the only internationally validated tool to evaluate dyspepsia in terms of both frequency and severity of the symptoms. It includes 8 questions that encompass dyspeptic symptoms and another on the most bothering symptom for patients (Additional file 1, 2 and 3).

The questionnaire has been improved in its short version, the choice of this study, and it includes the 4 main questions posed by general practitioners and gastroenterologists in the evaluation of dyspepsia. Finally, the GERD impact scale (GIS) was used for the evaluation of gastroesophageal reflux disease. It is an easily applicable questionnaire consisting of 4 questions that evaluate the frequency and intensity of the symptoms related to GERD and the treatment development [2,11-13] (Additional file 1, 2 and 3).

The complementary evaluation of the dyspeptic disease was made by esophagogastroduodenoscopy using an Olympus GIF-180 gastrointestinal videoscope (USA) along with the analysis of *Helicobacter pylori* (*H.pylori*) infection through a serological or urease test performed in those patients who could not undergo a biopsy.

Endoscopies performed up to 6 months prior to the interview were considered valid for use in this research. Those individuals who did not fit in this criterion were requested to undergo a new procedure specifically for the study’s sake.

The questionnaires were applied during dyalisis sessions for hemodyalisis group and in the gastroenterology department for the other ones. Every interview took twenty minutes during the hemodyalisis session and the outpatient visits.

### Statistical analysis

The sample size was made in order to obtain a test power of 80% and a difference of 35% between groups with significance level of 5% based on a two-tailed model. The sample number was of 31 patients.

Thirty- five patients were recruited for each group with no loss in the hemodyalisis group and with, respectively, 3 and 4 patients excluded because of the no realization of the endoscopies.

The continuous variables used for the analysis of the patient’s profile included the values of creatinine and urea, age and creatinine clearance calculated by the application of the simplified MDRD formula. The categorical variables applied encompassed the use of proton pump inhibitors, the use of acetylsalicylic acid, gender, the presence of *H.pylori*, hypertension, diabetes and smoking defined as a regular cigarette consumption.

For the quantitative variables the analysis was obtained by mean and standard deviation calculation. For the qualitative variables the absolute and relative frequencies were used.

For the comparison of means between two groups Student’s *t*-test was used, and when the supposition of data normality was rejected the nonparametric Mann–Whitney test was applied.

For a three-group comparison the two-way ANOVA was used by the Bonferroni test, and when the supposition of data normality was rejected, the nonparametric Kruskal-Wallis test was applied with Dunn’s pairwise comparison test.

The evaluation of homogeneity between proportions was performed using the chi-square or the Fisher exact test when there were expected frequencies less than five.

In order to determine factors associated with events, the multivariate logistic regression model was used. The significance level applied for the tests was 5%. The statistical package used in this study was the SPSS, version 17.0, for Windows (Microsoft-USA).

## Results

As shown in Table 1, in relation to the characterization of GFR, the groups differed in terms of creatinine, urea and estimated clearance values calculated using the simplified MDRD formula with p < 0.001. The groups also differed concerning the use of acetylsalicylic acid, more frequent in the hemodialysis group, and the use of proton pump inhibitors (PPI), with a higher frequency in the hemodialysis group and a lower frequency in the control group (74.29% and 29.03% respectively).

**Table 1:** Assocation between the groups with the continuous and categorical variables studied

	**Hemodyalisis**	**CKD**	**Control**	**p**
	**N = 35**	**N = 32**	**N = 31**	
Age (years)	46 ±11.59	52.53 ± 12.46	44.09 ± 13.32	p = 0.021^β^
Men (%)	37.14	31.25	19.35	p = 0.278^θ^
Diabetes (%)	17.14	21.88	35.48	p = 0.205^θ^
Hypertension (%)	100	75	12.90	p < 0.001^θ^
*H*.*pylori *(%)	28.57	40.63	22.58	p = 0.283^θ^
Smoking (%)	0	12.50	6.45	p = 0.076^φ^
Urea (mg/dl)	156.53 ± 46.16	57.10 ± 27.69	29.6 ± 7.38	p < 0.001^β^
GFR (ml/min)	7.02 ± 7.07	61.81 ± 29.76	128.35 ± 51.47	p < 0.001^α^
Creatinine (mg/dl)	11.32 ± 4.24	1.47 ± 0.78	0.75 ± 0.17	p < 0.001^β^
Caucasian (%)	51.48	75	87.1	p = 0.005^θ^
AAS (%)	74.29	15.63	6.45	p < 0.001^θ^
PPI* (%)	74.29	31.25	29.03	p < 0.001^θ^
Abnormal Endoscopy (%)	28.57	12.50	16.13	p = 0.021^θ^

α – Kruskal-Wallis non parametric test.

β- Bonferroni’s test.

θ- Chi-square test.

φ- Fisher’s exact test.

*- Proton Pump Inhibitor.

As to the evaluation of functional dyspepsia, organic dyspepsia and GERD outcomes, the groups were compared using the chi-square test (Table 2). The presence of organic dyspepsia was the only one with statistical difference among the three groups, with p = 0.014, and a higher ratio in the control group (45.24%). This result suggests that patients with better glomerular function rate (GFR) have higher chances of presenting dyspepsia.

**Table 2:** Presence of functional dyspepsia, organic dyspepsia and GERD among the groups

	**Hemodyalisis**	**CKD**	**Control**	p*
	**N = 35**	**N = 32**	**N = 31**	
Functional Dyspesia (%)	31.03	31.03	37.93	0.669
Dyspepsia (%)	21.43	33.33	45.24	0.014
GERD(%)	26.32	28.95	44.74	0.075

* Chi-square test.

Table 3 shows the multivariate logistic regression model with the selection of age, creatinine and urea variables. There was a significant difference for age in the functional dyspepsia and dyspepsia outcomes (p = 0.006 and p = 0.043 respectively). Creatinine levels were statistically significant for the GERD group (p = 0.033). Despite the fact creatinine is correlated with GERD in this analysis, it did not show any relation with the functional dyspepsia and dyspepsia outcomes. Likewise, the urea value showed no relation with the mentioned outcomes.

**Table 3:** Multivariate logistic regression model associating age, creatinine and urea values with the presence of functional dyspepsia, organic dyspepsia and GERD

	**Functional Dyspepsia**	**Organic Dyspepsia**	**GERD**
Age (years)	p = 0.006	p = 0.043	p = 0.363
Creatinine(mg/dl)	p = 0.453	p = 0.102	p = 0.033
Urea (mg/dl)	p = 0.774	p = 0.503	p = 0.185

Table 4 shows the sorting of patients according to the outcome regardless of group they belonged to. The characteristic of creatinine and GFR observed in Table 4 was found significant for dyspepsia (p = 0.021 and 0.009 respectively) and for GERD (p = 0.009 and 0.015 respectively). Our study suggests that patients with better GFR have higher chances of presenting GERD.

**Table 4:** Univariate analysis of the presence of functional dyspepsia, organic dyspepsia and GERD with the continuous and categorical variables studied

	**Functional Dyspepsia + N = 29**	**Functional Dyspepsia – N = 69**	**p**	**Organic Dyspepsia + N = 42**	**Organic Dyspepsia – N = 56**	**p**	**GERD + N = 38**	**GERD – N = 60**	**p**
Diabetes (%)	13.79	28.99	0.110^θ^	28.57	71.43	0.415^θ^	28.95	21.67	0.414^θ^
Age (years)	42.06 ± 13.4	49.82 ± 11.94	0.006^β^	44.92 ± 14.36	49.48 ± 11.28	0.094^β^	46.73 ± 14.08	48.03 ± 12.05	0.628^β^
Men (%)	20.69	33.33	0.210^θ^	21.43	35.71	0.125^θ^	15.79	38.33	0.017^θ^
Hypertension (%)	58.62	66.67	0.440^θ^	52.38	73.21	0.033^θ^	52.63	71.67	0.055^θ^
*H.pylori *(%)	27.59	31.88	0.673^θ^	28.57	32.14	0.704^θ^	26.32	33.33	0.462^θ^
Smoking (%)	3.45	7.25	0.666^φ^	11.9	1.79	0.081^φ^	7.89	5	0.674^φ^
Urea (mg/dl)	74.75 ± 56.45	87.76 ± 66.88	0.361^β^	69.59 ± 54.45	94.65 ± 69.79	0.055^β^	70.52 ± 54.05	92.39 ± 68.6	0.099^β^
GFR (ml/min)	78.72 ± 3.18	56.81 ± 52.99	0.152^α^	77.95 ± 58.07	52.30 ± 59.80	0.009^α^	79.84 ± 64.21	52.81 ± 55.41	0.015^α^
Creatinine (mg/dl)	3.92 ± 4.89	5.11 ± 5.79	0.336^β^	3.27 ± 4.68	5.87 ± 5.91	0.021^β^	3.08 ± 4.03	5.82 ± 6.11	0.009^β^
Caucasian (%)	72.41	69.57	0.779^θ^	69.05	71.43	0.798^θ^	76.32	66.67	0.307^θ^
AAS (%)	27.59	36.23	0.408^θ^	21.43	42.86	0.026^θ^	21.05	41.67	0.035^θ^
PPI (%)	51.72	48.28	0.454^θ^	42.86	48.21	0.598^θ^	50	43.33	0.518^θ^
Abnormal Endoscopy (%)	20.69	18.84	0.832^θ^	26.19	14.29	0.141^θ^	23.68	16.67	0.391^θ^

α –Mann–Whitney non parametric test.

β- Student’s *t* test.

θ- Chi-square test.

φ- Fisher’s exact test.

## Discussion

There are few works relating the worsening of kidney function and the developing of dyspepsia. In our study it was possible to establish a difference between the groups in relation to the GFR levels. This enabled us, through the application of questionnaires, to correlate the alterations in creatinine values and the presence or absence of the mentioned outcomes.

Dyspeptic alterations in patients with renal disease have long been studied. Different models have been developed for a better comprehension, and many physiopathological explanations have been found.

Patients with CKD are more likely to present gastric mucosal injuries than the population in general owing to local or systemic circulatory insufficiency, hypergastrinemia and higher levels of ammonia and inflammation [14].

However, whether the presence of abnormalities in the gastric mucosa develops into disease with a positive correlation between the intense symptomatology and the presence of functional dyspepsia and GERD are questions that still puzzle researchers.

Strid et al. [15] investigated the relation between dyspeptic symptoms and patients in dialysis and pre-dialysis programs. Their findings are consonant with this study concerning the higher symptomatology among patients in hemodialysis. Besides, a lower quality of life was observed among dialytic patients with more symptoms [15].

Still, there are very few reports that compare patients who undergo substitutive renal therapy with those who do not. Guz et al. [16] compared the existence of emptying delay in patients in hemodialysis and in peritoneal dialysis without finding any difference taking the dialytic method into consideration [16,17].

Another factor associated with dyspepsia in patients with CKD is the infection by *Helicobacter pylori*. We did not detect any difference in the *H. pylori* infection between groups or when only its presence or absence was compared in the outcomes. The infection prevalence among patients undergoing conservative treatment or dialysis is lower than in the population as a whole [14].

The pH elevation of the atrophic gastric mucosa as a result of the persistent inflammatory status, uremia, and the wide use of antibiotics on account of the higher risk of infections they have, are some of the several factors that justify this lower rate of infection by *H.pylori*[14].

When only the outcomes were taken into consideration, there was no difference between groups in relation to the presence of functional dyspepsia and GERD. Nevertheless, the presence of dyspepsia, according to the evaluation by the short-form Leeds dyspepsia questionnaire, was statistically significant, with a higher rate of dyspeptic patients in the control group. This result conflicted with the one found in the other studied series, which showed prevalence of dyspepsia in the order of 25 to 75% of patients with CKD [18].

Yet, the protector factor of PPI use in its genesis was not significant in this analysis.

The worsening of the functional stage of CKD, according to the proposed model in this study, showed an inverse correlation with the development of GERD. The GERD group presented lower mean values of creatinine and clearance estimated by the MDRD formula.

GERD is multifactorial, and it is related to hypochlorhydria and hypergastrinemia in patients with CKD. Nevertheless, an opposite result was observed, which was not related to the use of PPI either.

However, despite the hypochlorhydria, we believe that the gastric alterations found in CKD are not acid-related, and this group presents unknown specific factors which make such patients more susceptible to the development of GERD and dyspepsia [19,20].

There were some limitations to this study. Among them, there was the impossibility to longitudinally follow up the patient so that we could identify possible triggering factors for dyspepsia and reflux in a timely manner due to the high mortality rate associated to hemodialysis.

In conclusion, the worsening of creatinine levels in the proposed model did not establish itself as a risk factor for the onset of dyspepsia in its syndromic and functional dyspepsia forms, and it was inversely correlated with GERD. The use of PPI did not affect the onset of the outcomes. Intrinsic factors related to CKD may be involved in the genesis of dyspeptic symptoms.

## Competing interests

The authors declare that they have no competing interests.

## Authors’ contributions

MRB collected the data and made the interviews and statistical analysis. EZC is the senior researcher and conducted the study. Both authors read and approved the final manuscript.

## Supplementary Material

Additional file 1 Gis impact scale. Click here for file

Additional file 2 Leeds short form questionnaire. Click here for file

Additional file 3 Functional dyspepsia module. Click here for file
