# Supplementary material for: Dyspepsia among patients with chronic kidney disease: a cross sectional study
Source: Int Arch Med. 2013 Oct 20;6:43. doi: 10.1186/1755-7682-6-43 (PMC4016304; doi:10.1186/1755-7682-6-43)
Supplement: Additional file 1 — Gis impact scale. [file 1755-7682-6-43-S1.pdf]

GIS IMPACT SCALE

In the past week...	Daily	Often	Sometimes	Never
1. How often have you had the following symptoms:				
a. Pain in your chest or behind the breastbone?	○	○	○	○
b. Burning sensation in your chest or behind the breastbone?	○	○	○	○
c. Regurgitation or acid taste in your mouth?	○	○	○	○
d. Pain or burning in your upper stomach?	○	○	○	○
e. Sore throat or hoarseness that is related to your heartburn or acid reflux?	○	○	○	○
2. How often have you had difficulty getting a good night's sleep because of your symptoms?	○	○	○	○
3. How often have your symptoms prevented you from eating or drinking any of the foods you like?	○	○	○	○
4. How frequently have your symptoms kept you from being fully productive in your job or daily activities?	○	○	○	○
5. How often do you take additional medication other than what the physician told you to take (such as Tums, Rolaids, Maalox)?	○	○	○	○
