# Supplementary material for: Dyspepsia among patients with chronic kidney disease: a cross sectional study
Source: Int Arch Med. 2013 Oct 20;6:43. doi: 10.1186/1755-7682-6-43 (PMC4016304; doi:10.1186/1755-7682-6-43)
Supplement: Additional file 2 — Leeds short form questionnaire. [file 1755-7682-6-43-S2.pdf]

LEEDS SHORT FORM QUESTIONNAIRE

Please answer both parts of each question

Patient ID:<br><br>Date:	**A**<br>**How often have you *had* this symptom over the last 2 months?**<br>Tick only one box per question.	**B**<br>**How often *has this symptom interfered with your normal activities (eating, sleeping, work, leisure)* over the last 2 months?**<br>Tick only one box per question
**1. Indigestion**<br>Indigestion is a pain or discomfort in the upper abdomen. 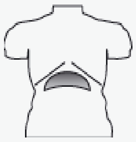	☐ Not at all<br>☐ Less than once a month<br>☐ Between once a month and once a week<br>☐ Between once a week and once a day<br>☐ Once a day or more	☐ Not at all<br>☐ Less than once a month<br>☐ Between once a month and once a week<br>☐ Between once a week and once a day<br>☐ Once a day or more
**2. Heartburn**<br>Heartburn is a burning feeling behind the breastbone. 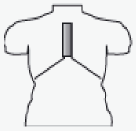	☐ Not at all<br>☐ Less than once a month<br>☐ Between once a month and once a week<br>☐ Between once a week and once a day<br>☐ Once a day or more	☐ Not at all<br>☐ Less than once a month<br>☐ Between once a month and once a week<br>☐ Between once a week and once a day<br>☐ Once a day or more
**3. Regurgitation**<br>Regurgitation is an acid taste coming up into your mouth from your stomach.	☐ Not at all<br>☐ Less than once a month<br>☐ Between once a month and once a week<br>☐ Between once a week and once a day<br>☐ Once a day or more	☐ Not at all<br>☐ Less than once a month<br>☐ Between once a month and once a week<br>☐ Between once a week and once a day<br>☐ Once a day or more
**4. Nausea**<br>Nausea is a feeling of sickness without actually being sick.	☐ Not at all<br>☐ Less than once a month<br>☐ Between once a month and once a week<br>☐ Between once a week and once a day<br>☐ Once a day or more	☐ Not at all<br>☐ Less than once a month<br>☐ Between once a month and once a week<br>☐ Between once a week and once a day<br>☐ Once a day or more

5. Which, if any, of these symptoms has been the most troublesome to you in the last 2 months?

Please tick one box only

☐ Heartburn
☐ Regurgitation
☐ Indigestion
☐ Nausea
☐ None of these have troubled me
