# Supplementary material for: Dyspepsia among patients with chronic kidney disease: a cross sectional study
Source: Int Arch Med. 2013 Oct 20;6:43. doi: 10.1186/1755-7682-6-43 (PMC4016304; doi:10.1186/1755-7682-6-43)
Supplement: Additional file 3 — Functional dyspepsia module. [file 1755-7682-6-43-S3.pdf]

Functional Dyspepsia Module		
1. In the last 3 months, how often did you have pain or discomfort in the middle of your chest (not related to heart problems)?	⓪ Never<br>① Less than one day a month<br>② One day a month<br>③ Two to three days a month<br>④ One day a week<br>⑤ More than one day a week<br>⑥ Every day	
2. In the last 3 months, how often did you have heartburn (a burning discomfort or burning pain in your chest)?	⓪ Never<br>① Less than one day a month<br>② One day a month<br>③ Two to three days a month<br>④ One day a week<br>⑤ More than one day a week<br>⑥ Every day	
3. In the last 3 months, how often did you feel uncomfortably full after a regular-sized meal?	⓪ Never →<br>① Less than one day a month<br>② One day a month<br>③ Two to three days a month<br>④ One day a week<br>⑤ More than one day a week<br>⑥ Every day	***Skip to question 5***
4. Have you had this uncomfortable fullness after meals 6 months or longer?	⓪ No<br>① Yes	
5. In the last 3 months, how often were you unable to finish a regular size meal?	⓪ Never →<br>① Less than one day a month<br>② One day a month<br>③ Two to three days a month<br>④ One day a week<br>⑤ More than one day a week<br>⑥ Every day	***Skip to question 7***
6. Have you had this inability to finish regular size meals 6 months or longer?	⓪ No<br>① Yes	
7. In the last 3 months, how often did you have pain or burning in the middle of your abdomen, above your belly button but not in your chest?	⓪ Never →<br>① Less than one day a month<br>② One day a month<br>③ Two to three days a month<br>④ One day a week<br>⑤ More than one day a week<br>⑥ Every day	***Skip remaining questions***
8. Have you had this pain or burning 6 months or longer?	⓪ No<br>① Yes	
9. Did this pain or burning occur and then completely disappear during the same day?	⓪ Never or rarely<br>① Sometimes<br>② Often<br>③ Most of the time<br>④ Always	

10. Usually, how severe was the pain or burning in the middle of your abdomen, above your belly button?	① Very mild<br>② Mild<br>③ Moderate<br>④ Severe<br>⑤ Very severe	
11. Was this pain or burning relieved by taking antacids?	⓪ Never or rarely<br>① Sometimes<br>② Often<br>③ Most of the time<br>④ Always	
12. Did this pain or burning usually get better or stop after a bowel movement or passing gas?	⓪ Never or rarely<br>① Sometimes<br>② Often<br>③ Most of the time<br>④ Always	
13. How often was this pain or discomfort relieved by moving or changing positions?	⓪ Never or rarely<br>① Sometimes<br>② Often<br>③ Most of the time<br>④ Always	
14. In the last 6 months, how often did you have steady pain in the middle or right side of your upper abdomen?	⓪ Never →<br>① Less than one day a month<br>② One day a month<br>③ Two to three days a month<br>④ One day a week<br>⑤ More than one day a week<br>⑥ Every day	***Skip remaining questions***
15. Did this pain last 30 minutes or longer?	⓪ Never or rarely<br>① Sometimes<br>② Often<br>③ Most of the time<br>④ Always	
16. Did this pain build up to a steady, severe level?	⓪ Never or rarely<br>① Sometimes<br>② Often<br>③ Most of the time<br>④ Always	
17. Did this pain go away completely between episodes?	⓪ Never or rarely<br>① Sometimes<br>② Often<br>③ Most of the time<br>④ Always	
18. Did this pain stop you from your usual activities, or cause you to see a doctor urgently or go to the emergency department?	⓪ Never or rarely<br>① Sometimes<br>② Often<br>③ Most of the time<br>④ Always	

B1. Functional Dyspepsia

Diagnostic criteria*

Must include:

1. One or more of:
    a. Bothersome postprandial fullness
    Uncomfortably full after regular sized meal, more than 1 day/week (question 3>4)
    Onset more than 6 months ago (question 4=1)
    b. Early satiation
    Unable to finish regular sized meal, more than 1 day/week (question 5 >4)
    Onset more than 6 months ago. Yes. (question 6=1)
    c. Epigastric pain
    Pain or burning in middle of abdomen, at least 1 day/week (question 7>3)
    Onset more than 6 months ago. Yes. (question 8=1)
    d. Epigastric burning
    (This criterion is incorporated in the same question as epigastric pain)

AND

1. No evidence of structural disease (including at upper endoscopy) that is likely to explain the symptoms
    No question.

* Criteria fulfilled for the last 3 months with symptom onset at least 6 months prior to diagnosis

Yes. *(question 8=1)*

B1a: Postprandial Distress Syndrome (PDS)

Diagnostic criteria*

Must include all of the following:

1. Bothersome postprandial fullness, occurring after ordinary sized meals, at least several times per week
    Uncomfortably full after regular sized meal, more than 1 day/week (question 3>4)
2. Early satiation that prevents finishing a regular meal, at least several times per week
    Unable to finish regular sized meal more than 1 day/week (question 5>4)

* Criteria fulfilled for the last 3 months with symptom onset at least 6 months prior to diagnosis

Requires a “Yes” to both. (question 4=1) & (question 6=1)

B1b: Epigastric Pain Syndrome (EPS)

Diagnostic criteria*

Must include all of the following:

1. Pain or burning localized to the epigastrium, of at least moderate severity at least once per week
    Pain or burning in middle of abdomen, at least 1 day/week (question 7>3)
    Pain is at least moderate severity (question 10>2)
2. The pain is intermittent
    Pain or burning often disappears completely in the same day (question 9>1)
3. Not generalized or localized to other abdominal or chest regions

Chest pain occurs once a month or less often (question 1 <3)

Heartburn occurs once a month or less often (question 2 <3)

4. Not relieved by defecation or passage of flatus

   Never or rarely gets better after defecation (question 12=0)

5. Not fulfilling criteria for biliary pain
6. Criteria fulfilled for the last 3 months with symptom onset at least 6 months prior to diagnosis

   Yes. (question 8 =1)

E. Functional Gallbladder and Sphincter of Oddi Disorders (for exclusion)

Diagnostic criteria*

Must include episodes of pain located in the epigastrium and/or right upper quadrant

Steady pain which may occur less than once per month (question 14>0)

AND <u>all</u> of the following:

1. Episodes lasting 30 minutes or longer

   At least often (question 15>1)

2. Recurrent symptoms occurring at different intervals (not daily)

   At least often (question 17>1)

3. The pain builds up to a steady level

   At least often (question 16>1)

4. The pain is moderate to severe enough to interrupt the patient's daily activities or lead to an emergency department visit

   At least often (question 18>1)

5. The pain is not relieved by bowel movements

   Never or rarely. (question 12=0)

6. The pain is not relieved by postural change

   Never or rarely. (question 13=0)

7. The pain is not relieved by antacids

   Never or rarely. (question 11=0)

8. Exclusion of other structural disease that would explain the symptoms.

   No question.
